# Exploring Perceptions of Age Friendliness Across Different Campus Constituent Groups

**DOI:** 10.1093/geroni/igab046.042

**Published:** 2021-12-17

**Authors:** Celeste Beaulieu, Nina Silverstein, Lauren Bowen, Susan Whitbourne, Joann Montepare

**Affiliations:** 1 University of Massachusetts Boston, Boston, Massachusetts, United States; 2 University of Massachusetts, Boston, University of Massachusetts Boston, Massachusetts, United States; 3 Lasell University, Newton, Massachusetts, United States

## Abstract

College campuses are typically considered as environments for adults ages 18-24, even though campuses are comprised of faculty, staff, students, and lifelong learners of all ages. Each group may experience the campus environment differently due to their differing roles. Faculty, staff and students from 21 participating designated Age Friendly Universities across the country answered survey questions on age friendliness, AFU awareness, and on campus practice items. Crosstab analyses show that constituent groups are equally aware of their university as an AFU (6% of each group). Students perceived their university as more age friendly (M=3.47, SD=0.73) compared to faculty and staff, the latter having the lowest perceived friendliness (M=3.27, SD=0.63). Specific age friendly practices show that staff members had markedly different perceptions of the institution’s age friendly practices. AFUs need to consider higher education environments as workplaces as well as learning centers to make policies age friendly for all groups.

